# A conceptual model for co‐developing a culturally tailored intervention for Latina immigrant caregivers of children with disabilities

**DOI:** 10.1002/ajcp.12789

**Published:** 2025-02-09

**Authors:** Yolanda Suarez‐Balcazar, Amy Pei‐Lung Yu, Stephany Brown, Jasmine Brown‐Hollie, Adriana Crostley, Deborah Parra‐Medina, Mariela Saenz, Mansha Mirza, Aileen Velasquez, Sandra Vanegas, Sandy Magaña

**Affiliations:** ^1^ University of Illinois Chicago Chicago Illinois USA; ^2^ University of Texas at Austin Austin Texas USA; ^3^ Autism Society of Texas Austin Texas USA; ^4^ University of Colorado Anschutz Medical Campus Aurora Colorado USA

**Keywords:** culturally tailored interventions, disability, Latinx immigrants, promotoras

## Abstract

The growing diversity of the U.S. population, partly due to immigration, has called attention to scholars and practitioners to attend to immigrants' cultural beliefs, values, and ways of doing when designing interventions to promote health and wellbeing. In this paper, we propose a contextual and dynamic model for co‐developing a culturally tailored intervention with the community to advance equity and empowerment of Latinx immigrant caregivers of children with intellectual and developmental disabilities (IDD). Grounded in the literature and voices of the community, the proposed model includes six interactive dimensions (**LARREDS**) that guided the development of the *PODER Familiar* intervention described here. These include **l**anguage and linguistic preferences; **a**ccessibility factors; **r**eflecting the group's values, ways of thinking and doing; **re**flecting generational differences; **d**imensions of delivery and learning style; and the **s**ocial, ecological, and cultural environment. Informed by principles of family engagement, the model also includes eight strategies for engaging caregivers throughout the intervention. The conceptual model was co‐developed with promotoras who also provided input on the *PODER Familiar* intervention. While describing the model in action, we highlight the voices of the promotoras. The implications of culturally tailored interventions and the application of the model to designing interventions for other migrant populations are discussed.

## Highlights

- The LARREDS model consist of specific dimensions and strategies for developing culturally tailored interventions.
- PODER Familiar is a culturally tailored intervention designed to promote the health and wellbeing of Latina immmigrant caregivers.
- Migrant communities must be involved in co‐developing culturally tailored interventions.

The growing diversity of the U.S. population calls for scholars and practitioners alike to attend to the cultural context of the population of interest by embracing the values, beliefs, and ways of thinking and doing when designing health promotion interventions. About 41.1% of the U.S. population is of diverse ethnic and racial backgrounds, which include Asian, Black, Latinx, Native American/Pacific Islander, or multiracial. It is projected that by 2040, nearly half of the U.S. population will be from diverse backgrounds other than White (United States Census Bureau, 2023a), and by 2060 or sooner, almost one in five individuals in the U.S. population will be foreign‐born (United States Census Bureau, 2023b) Approximately 44 million people in the U.S. identify as first‐generation immigrants (Vespa et al., 2020). One group that is growing significantly is the Latinx population, which in 2022 comprised 63.7 million people or 19% of the total population (United States Census Bureau, 2023a). One of every four children between the ages of 4–12 is from a Latinx background (National Center for Hispanic Research on Children and Families, 2024).

Latinx families of children with disabilities have been historically marginalized in the healthcare system (Bishop‐Fitzpatrick & Kind, 2017). Latinx immigrant caregivers often experience persistent health disparities and numerous systemic barriers and contextual stressors (e.g., language barriers, financial burden, discrimination, and social isolation) in accessing programs that could promote their health and wellbeing, and these disparities are exacerbated for families of children with intellectual and developmental disabilities (IDD) (Balcazar et al., 2020; Magaña et al., 2015; Suarez‐Balcazar, Viquez, et al., 2020).

Scholars argue that evidence‐based interventions and practices are often not developed in collaboration with diverse groups or tested with culturally diverse populations (Castro & Yasui, 2017; Castro et al., 2010). As such, several entities, including the Centers for Disease Control and Prevention (CDC, 2023 see https://www.cdc.gov/nccdphp/dnpao/state-local-programs/reach/index.htm), the National Institutes of Health, the U.S. Department of Health and Human Services, Office of Minority Health, and various scholars have emphasized the need to develop culturally tailored interventions to meet the needs of diverse populations (Joo & Liu, 2021; Lee et al., 2023; Magaña et al., 2020; Suarez‐Balcazar, 2020).

In this article, we propose a model for developing culturally tailored health promotion interventions. We describe the model, LARREDS, and its application to the development of *PODER Familiar* (Spanish for Family Power*)*, a health promotion program that utilizes the Promotoras de Salud model (Centers for Disease Control and Prevention, 2019), to promote the health and wellbeing of Latinx immigrant families of children with IDD. This work is unique because our community advisory board and promotoras (Community Health Workers) provided ongoing input on the *PODER Familiar* intervention, its accompanying curriculum, and the LARREDS model itself. Furthermore, the LARREDS model contributes to the limited existing literature on the frameworks for developing culturally tailored interventions.

In this article, we used Latinx as an inclusive term to refer to people of Mexican, Central, or South American origins and from diverse genders. We used Latina when referring to female caregivers of children with disabilities. Other terms used in the literature include Hispanics (traditionally used by the federal government, U.S. Census data, and national databases) and Latinos (mostly preferred by community stakeholders). We kept the term Latinos when used explicitly by a promotora involved in *PODER Familiar*, as described later in this paper.

## CULTURALLY TAILORED INTERVENTIONS

For community health promotion interventions to be effective, they must be culturally responsive to the target group by reflecting their values, beliefs, and ways of thinking and doing (Bernal, 2006; Magaña et al., 2020). Research indicates that community interventions catering to a cultural group's specific needs are significantly more effective than generic interventions (Torres‐Ruiz et al., 2018) and more likely to promote health equity, reduce health disparities, build on community strengths, and optimize community engagement (Barrio & Yamada, 2010; Bernal, 2006; Joo & Liu, 2021; Lee et al., 2023; Rhodes et al., 2017; Suarez‐Balcazar et al., 2016). Most community interventions that are tailored for specific cultural groups have been limited to surface adaptations, which typically involve translating the protocol and materials to the preferred language of the immigrant group, depicting people and foods from the community, and/or hiring and training staff from the target community to deliver the intervention (Barrio & Yamada, 2010; Resnicow et al., 2009). While these efforts improve comprehension of the intervention, often these interventions stay at the surface level, overlooking accessibility, social and familial context, and sometimes generational differences. This oversight can hinder the implementation process, desired outcomes, and sustainability of interventions (Castro et al., 2010).

Culturally tailored interventions are defined as programs that include components that are aligned with and reflect the target group's values, ideas, beliefs, ways of doing, ways of thinking, and ways of being (Bernal, 2006; Castro et al., 2010). Culturally tailored interventions also consider the heterogeneity of the target population, ensuring that intervention components are inclusive and accessible to all participants, including people with disabilities. To achieve this, co‐developing the intervention with the community of interest through constant engagement is key (Lee et al., 2023; Magaña, 2021; Suarez‐Balcazar, 2020) as community members are vital in assessing community needs, priorities, and assets, framing the issue of concern, and addressing such concerns (Rhodes et al., 2017; Wallerstein, 2021). Strategies for promoting community engagement can involve extended periods of mutually beneficial collaboration with the community, establishing community advisory boards, including community leaders in the research team, and compensating community members for their time, among others (Fernández et al., 2023; Suarez‐Balcazar, 2020).

A few models and key concepts are available in the literature to guide and inform the process of culturally adapting and tailoring evidence‐based interventions (Barrio & Yamada, 2010; Bernal, 2006; Lee et al., 2023). However, most of this literature focuses on adapting existing evidence‐based interventions, and evidence on the effectiveness of these interventions remains limited. As such, Bernal and Adames (2017) question the conceptual, ethical, contextual, and methodological process of culturally adapting evidence‐based interventions. The authors argued that alternative methodologies are needed in prevention science. This article aims to illustrate the LARREDS model and propose it as a framework to guide the development of health promotion interventions for immigrant populations. The dimensions highlighted in the LARREDS model are particularly vital for designing interventions for populations at the intersections of multiple minoritized identities, such as immigrant families experiencing disability. Although some components in the model were derived from other models such as language preference and reflecting values and ways of doing, other factors such as accessibility and social context of the family are novel to this model.

## THE LARREDS MODEL

Our work was informed by Shaia's (2019) SHARP framework for equity interventions with marginalized communities, applicable to migrant populations. Shaia's SHARP framework emphasizes five critical components: *structural oppression*, *historical context, analysis of role, reciprocity, and power*. These framework components were valuable in understanding the Latinx population in the U.S.

Latinx communities, as an immigrant group, have experienced structural oppression and a history of exclusion and marginalization in the U.S. (Buckingham & Brodsky, 2020; Fernández et al., 2023; Torres et al., 2022). For instance, with the Treaty of Guadalupe Hidalgo in 1848, the U.S. government took possession of large parts of Mexican territory, making many who were once Mexican citizens minorities in their own land (Adames & Chavez‐Dueñas, 2017). These displaced individuals became known as Mexican Americans. Current Latinx communities consist of these earlier generations of Mexican Americans and first and second‐generation immigrants from various Latin American countries, who have also experienced social and economic marginalization and have been regular targets of xenophobia and racism (Fernández et al., 2023). Recognizing and leveraging strengths and moving away from a deficit approach has also been critical to studying the Latinx population (Suarez‐Balcazar et al., 2021).

In developing the *PODER Familiar* intervention, we intentionally developed content that built on community strengths, such as familismo, resiliency, and the cultural importance of kinship networks (see Adames & Chavez‐Dueñas, 2017). Analysis of role, reciprocity, and power was also essential to the development of *PODER Familiar*, as illustrated by the involvement of promotoras in delivering the intervention. Our promotoras all identified as Latina caregivers of children with IDD who share similar parenting experiences and cultural and ethnic backgrounds as our program participants, some residing in mostly Latinx communities, thus moving away from a traditional power differential between the participant and researcher or the client and service provider. The promotoras also played a critical role in developing the intervention and the LARREDS model. They provided ongoing feedback on the intervention program components, which was documented through biweekly meetings with the investigators. Based on the literature of existing models (see Bernal & Adames, 2017; Castro et al., 2010; Joo & Liu, 2021; Lee et al., 2023) and our work with the Latinx community over the last 25 years, we proposed a contextual and dynamic model for culturally tailored interventions, developed from the ground up in collaboration with the Latinx community, particularly our promotoras and Latina caregivers.

**Figure 1:**
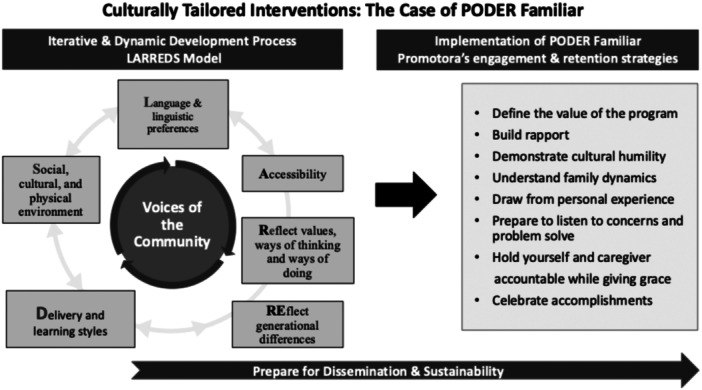
*Culturally Tailored Interventions: The LARREDS Model*.

The LARREDS model was developed with a focus on capitalizing on the community's shared knowledge and expertise (Baumann & Cabassa, 2020). As shown in Figure 1, the processes organic to the LARREDS model are dynamic and require ongoing collaboration and meaningful engagement with the target community so that their voices are valued and heard (Baumann & Cabassa, 2020; Fernández et al., 2023; Rhodes et al., 2017; Suarez‐Balcazar, 2020).

The model illustrates six dimensions that facilitated intervention development. At the core of the six interactive dimensions, that influence and intertwined with each other, are the voices of the community. Such voices included the caregivers, promotoras, and members of our project's Community Advisory Board who provided extensive suggestions on developing the model and its six dimensions. These six dimensions include (a) **l**anguage and linguistic preferences; (b) **a**ccessibility factors; (c) reflect values, ways of thinking, and ways of doing; (d) reflect generational differences; (e) **d**imensions of delivery and learning styles; and (e) **s**ocial, cultural, and physical environment.

These six dimensions are complemented by eight engagement and retention strategies relevant to sustaining the engagement of participants in culturally tailored interventions for immigrant families experiencing disability. These eight strategies were identified by one of our promotoras (coauthor A.C.) based on her vast experience supporting families and the extensive literature on family engagement. The eight strategies included: defining the value of the program, building rapport, demonstrating cultural humility, understanding family dynamics, drawing from personal experience, preparing to listen and problem‐solve, holding yourself and the caregiver accountable while giving grace and celebrating accomplishments. Notably, these engagement dimensions are consistent with the extensive literature on family engagement principles and frameworks. For instance, Ball et al. (2020) developed a framework that includes providing support, establishing rapport, establishing trust, and empowering families. Similarly, Clark‐Louque and Sullivan (2020) described the seven evidence‐based principles of family engagement, including communication, collaboration, compassion‐caring, culture, connection, community, and collective responsibility. Castro and Yasui (2017) also pointed to the need to attend to engagement strategies as they may influence sustainability.

The PODER Familiar intervention serves as an example of the LARREDS model in application. In the next section, we illustrate the six dimensions of LARREDS and the eight engagement strategies through PODER familiar while centering the voices of the promotoras.

## PODER FAMILIAR

*PODER Familiar* is an intervention grounded in principles of community‐engaged research and culturally tailored health promotion interventions for Latinx families (Magaña et al., 2021; Suarez‐Balcazar, 2020), Bandura's self‐efficacy theory (Bandura, 1977), and the Social Ecological Model (McLeroy et al., 1988). At the center of the intervention development were the voices of Latina caregivers of children with IDD. To create a climate of co‐learning, we formed a community advisory board at the beginning of the project, and we recruited and trained promotoras to co‐develop and deliver the intervention. *PODER Familiar* was also informed by two existing evidence‐based interventions that were developed in collaboration with Latina caregivers of children with disabilities by members of our team. The first intervention, *Caring for Myself*‐(Magaña et al., 2015), was designed to promote the wellbeing of the caregiver‐‐and *th*e second intervention, *Familias Saludables* (Suarez‐Balcazar et al., 2016, 2023) was designed to promote the health of the whole family of a child with IDD. The integration of these two programs led to the initial draft of the *PODER Familiar* curriculum. We sought feedback on the draft curriculum from our community advisory boards consisting of Latinx disability advocates, caregivers of young adults with disabilities, and professionals serving the Latinx community of people with disabilities and their families. After incorporating their feedback, we revised and piloted the curriculum with our study participants in Texas and Illinois. Throughout the pilot testing phase, the promotoras took note of recommendations for improving the curriculum and its delivery, as well as informed components of the model. This feedback was communicated to the local project director and included in the final product. Their feedback included content (e.g., using plain language, using cultural terms, and cultural examples of foods and meals); curriculum organization and format (e.g., for instance, adding more visuals, bullet points, and more short cases and stories); suggestions for adding resources on mental health, seeking mental health support, and examples of ways to manage stress; and logistical issues (e.g., managing zoom, sharing the screen).

*PODER Familiar* consisted of ten individual sessions and three group sessions that aimed to promote a healthy lifestyle and positive health outcomes among Latinx families of children with IDD. Based on the two evidence‐based interventions used to develop *PODER Familiar* and existing literature on health promotion (Magaña et al., 2014; Magaña et al., 2015; Torres‐Ruiz et al., 2018), the individual sessions were developed under four main themes: caregivers' wellbeing, nutrition and dietary routines, physical activity, and managing home and community environment. Overall, to facilitate learning, the *PODER Familiar* curriculum manual included plenty of visuals, short stories, and concrete, relatively simple strategies for making lifestyle changes.

A critical component of *PODER Familiar* was the incorporation of promotoras in co‐developing and delivering the intervention. This workforce has been widely employed in the field of community health and public health to effectively educate and support the health of residents in communities of color (Magaña et al., 2014). Promotoras are typically lay people from the community who are trained to provide health education and support, engage in health promotion efforts, and implement health or empowerment‐focused interventions (Hernandez‐Salinas et al., 2023; Magaña et al., 2020). However, besides the work of Magaña et al., (2014, 2015), few studies have incorporated promotoras in co‐developing and delivering interventions to promote the health and wellbeing of Latina immigrant caregivers of children with IDD.

Promotoras in *PODER Familiar* identified as Latina mothers or female family caregivers of a child with IDD, fluent in both Spanish and English and with experience in supporting other Latinx families (e.g., volunteering or working for community organizations that serve families of children with IDD). All promotoras were interviewed to ensure qualifications and commitment for the duration of the study and were compensated for their work. In total, we recruited five promotoras (three in Texas and two in Illinois), and all received 10 h of extensive training as a group alongside research staff. We mainly designed our training to focus on rapport‐building, cultural humility, professionalism, and, most importantly, roleplay delivering the intervention and problem‐solving to address potential challenges. Promotoras received ongoing training and feedback via individual meetings with project coordinators at both sites. This was critical to ensuring promotoras felt comfortable with the content and delivery before the first session.

## THE LARREDS MODEL IN ACTION

The voices and contributions of the promotoras are integrated throughout the next section as we describe the model in action. The stories from the promotoras below came from the analysis of the surveys completed online and ongoing feedback shared with the local project director.

### Language and linguistic preferences

One of the most important considerations when developing *PODER Familiar* was the caregivers' language and linguistic preferences. We made the intervention available in Spanish and English to accommodate language preferences among Latina immigrant caregivers. We also paid attention to health literacy. Thus, we used plain language as suggested by the CDC Plain Language Checklist (Centers for Disease Control and Prevention, 2019). Ongoing feedback on language usage, including dialects, was sought from caregivers and promotoras throughout the development process. To further customize the intervention, our promotoras adjusted their language of delivery depending on the caregiver's preference. One promotora from Texas indicated,

Some caregivers prefer to use English and Spanish interchangeably to describe precisely what they mean, so it is beneficial that we are bicultural and bilingual to truly understand these caregivers' needs and concerns.

We also observed differences in the preferred language used. Most of the caregivers in Illinois were first‐generation recent immigrants. They preferred to use Spanish, while several of the Texas caregivers were second‐generation immigrants and or had lived longer in the U.S. and preferred the intervention in English.

### Accessibility factors

Accessibility is often overlooked when designing and implementing health‐related programs, even though one in six Latinx individuals have a disability, and the majority live with their families (Balcazar et al., 2020). To develop an intervention that is inclusive and accessible, we considered our promotoras and the caregivers' feedback for ways to adapt physical activities and health promotion strategies for children with IDD. For example, many caregivers have children who display wandering behaviors, and it is often difficult for these families to feel safe when exercising outdoors. One promotora suggested reconsidering physical activity locations. For instance, try walking on a fenced running track instead of around the neighborhood. Another promotora from Texas shared,

I would always ask the caregivers what is accessible around them. Is there a park nearby? Is there a sidewalk? What are the barriers that stop them from exercising? From there, we would discuss how to overcome accessibility issues.

Promotoras also shared with caregivers how to create visual cards and visual prompts they could utilize at home to communicate with children with IDD with low verbal behavior.

### Reflecting values, ways of thinking, and doing

*PODER Familiar* integrated the values, behavioral preferences, spirituality, foods, and other items from different Latinx cultures. We also included short stories, discussions of Latinx myths and common sayings, metaphors, and Latinx ways of thinking and doing. Some examples included the use of a traffic light system to illustrate the process of goal setting (see Suarez‐Balcazar et al., 2022); a story that illustrates obtaining support from a local faith‐based organization given the strong sense of spirituality common in many Latinx families (see Suarez‐Balcazar et al., 2021), and focus on common Latinx values such as familism and collective decision making (see Adames & Chavez‐Dueñas, 2017). Further examples of considering Latinx ways of doing things and cultural values involved adaptations to MyPlate (https://www.myplate.gov/), where caregivers were asked to think of how to prepare a colorful tortilla or a colorful meal plate. Noteworthy, Latinx is a very heterogeneous group, and differences may be marked by preferences in food items, music, and activities, among other factors.

Research has shown that the Latinx community enjoys passing on cultural values and learning from stories (Bernal, 2006). We included several short stories in the curriculum to illustrate cultural values and families' challenges in maintaining a healthy lifestyle. The promotora discussed the short stories with the caregivers and identified strategies to address the challenges. Our promotoras reported that many of the caregivers from both sites were able to relate to the joy and hardships depicted in the stories, and they were able to connect on a deeper level through the discussions. A promotora from Illinois stated, “After reading one of the short stories, the caregiver replied, that is me in the story”. A Texas promotora shared similar sentiments, “one of my caregivers replied after reading a story. I am that mother in the story”. This was a common expression among caregivers. The short stories were valued and seemed to reflect ways of thinking for the caregivers.

### Reflecting generational differences

Existing models for developing culturally tailored interventions emphasize the need to consider values and cultural ways of doing and thinking. Yet, little attention has been placed on generational differences. Although generational differences can be illustrated under different ways of thinking and cultural values, it is essential to attend to this dimension when designing interventions targeting immigrants. Latinx immigrants often live in multigenerational households, reflecting generational and acculturation differences (Adames & Chavez‐Dueñas, 2017; Rodriguez et al., 2007). Caregivers in the *PODER Familiar* program comprised mostly first‐ and second‐generation immigrants. Per caregivers' feedback, the foods and stories illustrated in the curriculum depicted typical Mexican, Central, and South American culture and some North American influences. Our promotoras discussed different ways to tailor strategies with the caregivers as they attended to generational and acculturation differences. Promotoras were trained to brainstorm adaptations to strategies taught. For example, in a session that focused on physical activity, a promotora from Illinois shared.

I discussed with one of the caregivers that she could play salsa music and dance while cooking or doing the dishes, as she said she liked to dance but had little time to do so. Yet, she replied yes, but when her son with IDD was helping in the kitchen, she would play American rap music. Although she expressed that she doesn't like rap music, we discussed ways to alternate the music.

Furthermore, multigenerational households may reflect different ways of thinking. A promotora shared that the caregiver had difficulty explaining to her mother‐in‐law, who lived with them, why removing the fat from the meat was important as a healthier option as the mother‐in‐law explained that for generations, women in her family told her that the fat carries all nutrients. To overcome this issue, our promotoras brainstormed with the caregivers how to convey the program and the content to their family members so that all family members could buy into the need to engage in healthier behaviors.

### Dimensions of delivery and learning styles

We considered the format and ways to deliver the intervention as critical components to ensure effectiveness and sustainability. *PODER Familiar* was designed to be delivered by trained promotoras who share parenting experiences and cultural backgrounds similar to our target community. This shared experience is essential as many of our caregivers revealed that they have a hard time sharing their struggles with others but felt relieved and validated when sharing with the promotoras, who also has a child with IDD. In addition to individual sessions, we offered three group sessions for the caregivers to learn from one another. A promotora from Texas indicated, “Many parents were thankful for the group sessions because they can learn from each other about ways to get their child with IDD to consume healthy foods and how and where to find community resources for children with IDD.” These group sessions allowed the caregivers to exchange strategies, brainstorm, learn from, and support one another. At one of the group sessions in Illinois, caregivers exchanged contact information and shared resources. Evidence shows that the Latinx community relies heavily on learning from one another's experiences and seeking support from family members who share similar cultures and experiences (Adames & Chavez‐Dueñas, 2017). As indicated by our promotoras, caregivers expressed gratefulness for being able to connect with a Latina mother who has experience raising a child with IDD.

### Social, cultural, and physical environment

Contextual factors (i.e., social, cultural, and physical environment) of the caregivers can impact how they live their lives. As such, our curriculum strongly emphasized how these factors could serve as barriers or facilitators to promoting healthy routines and wellbeing. Our promotoras were also equipped to help caregivers with social and environmental issues with Zoom meetings and to be flexible in understanding the caregiver's environment. A promotora from Illinois shared,

One caregiver did not want to turn the lights on in the room where she was during the sessions, and I was having a hard time seeing her face and establishing eye contact. The participant expressed that her house was small and too crowded, and several people lived there, so she did not want the promotora to see the chaos.

In this case, the promotora assured her it was ok, made her feel comfortable, and respected the caregiver's decision. Another promotora discussed strategies for where and how to purchase fresh produce and healthy foods on a limited income with a caregiver. Another promotora instructor instructed the caregiver to turn on the video on her computer. While promotoras were trained to follow the curriculum session by session, including all activities and prompts, they were also instructed to respond to specific contextual issues and problem‐solve according to the context of the caregiver, as illustrated above.

### Implementation of PODER familiar: Promotoras' engagement and retention strategies

Complementary to the LARREDS model, we have included a list of promotoras' engagement and retention strategies. As indicated earlier, these strategies for family engagement have been discussed vastly in the literature (see Ball et al., 2020; Ford et al., 2016), and scholars have pointed to the need to attend to the process of engaging participants in the intervention (Castro & Yasui, 2017). One of our Texas promotoras (coauthor A.C.) outlined eight essential strategies to keep the caregivers motivated and engaged throughout the *PODER Familiar* intervention.

#### Define the value of the program

A.C. described the importance of conveying the program's value to the caregivers during the first session. She explained that,

Parents of children with disabilities often have many things on their plate, so it is hard for them to dedicate time to the program. I ensured they knew how important this program was for themselves and their family members. I know they will benefit tremendously from the program and see visible results if they make the time for it. As indicated by a promotora, to take care of my child with IDD and my family, I have to learn to take care of myself.

#### Build rapport with the caregiver

Building a genuine connection with caregivers and their families was essential. While it may seem trivial, starting every session with a warm check‐in question like “How was your week?” can make the caregiver feel like someone cares about them. Furthermore, taking the time to get to know the caregivers, the names of their children with IDD, and other family members is critical to a good relationship.

#### Demonstrate cultural humility

Given that Latinx culture is highly heterogeneous, promotoras must demonstrate cultural humility when interacting with caregivers. This means respecting the caregivers' culture, traditions, and ways of doing and thinking. A.C. shared,

There are differences in cultures and traditions even within the same country. For example, people from the north of Mexico prefer flour tortillas, whereas people from the south of Mexico prefer corn tortillas. And other families may not eat tortillas. We can't assume that because we are all Latinas, we all eat tortillas. We, as promotoras, must be aware of differences in subcultures and tailor the example's content to their preferences.

#### Understand family dynamics

As indicated above, many Latinx families often live in intergenerational households or with other extended family members. Thus, understanding the complexity of the family dynamics is critical to the caregivers' outcomes from the program. A.C. explained,

Latina caregivers often live with their parents or in‐laws, and sometimes, it is difficult for her to have a say in what to buy or make healthy meals. In some situations, these caregivers rely on their husbands to do the grocery shopping from time to time, and they might not want to read the labels [the *PODER Familiar* intervention taught caregivers how to read and interpret food labels]. I try to understand their family dynamics and how the caregivers can help their family members understand the contents of this program so that they, too, can be involved.

#### Draw from personal experience

One distinct aspect of our promotoras is that they, too, are caregivers of a child with IDD. This shared lived experience is powerful when promotoras connect with the caregivers when they know the person has been through similar experiences. A.C. described this experience as,

I felt that to have a genuine connection, I had to be very open and share things that I have gone through, including issues with mental health. Because of my openness to share, the caregivers were also willing to share their struggles.

#### Prepare to listen to concerns and solve problems

The caregivers in this program came from all walks of life and faced many challenges. A.C. provided an example of problem‐solving by sharing that,

One of the caregivers worked full‐time and had a child with high support needs, so it was challenging for her to have the time to make homemade nutritious meals. She would often feel discouraged that she could not reach her goals set for herself. We tried various ways, including creative ways to meal prep in advance so that she could make homemade meals quickly or use disposable plates, so she does not have to do the dishes. When these strategies also did not work, we decided to set a goal where she was still eating out, but she would look for better options on the menu. She began reaching her goals and became very motivated.

#### Hold yourself and the caregiver accountable while giving grace

To be fully engaged, promotoras and caregivers must hold themselves accountable. This means that they should consistently schedule weekly sessions with the caregivers and reschedule immediately if there are cancellations. A.C. shared the importance of assisting the caregivers in holding themselves accountable.

Mothers or female caregivers are often portrayed as selfless in the Latino culture, so it is tough for them to understand the concept of taking care of themselves so that they can take care of others. Initially, I had one caregiver only write down goals for her child with IDD. I could relate to that because, for years, I was only focused on my child's wellbeing but not myself. I explained to her the importance of keeping herself accountable so that she could regain strength in this journey.

#### Celebrate accomplishment

Celebrating accomplishments every step of the way is essential so that the caregivers remain motivated throughout the program. A.C. explained her experience by stating,

It is important to celebrate accomplishments, whether they are big or small. Many caregivers loved our discussions on recipes and brainstorming strategies for their children to eat various foods. They were always very excited to share what they had done and what worked and did not. They were also very encouraged when they reached their weekly goals and looked forward to achieving the following goals. Another caregiver was very isolated at first. Throughout the program, we set goals for building her social support network, and by the end, she had over a dozen new connections and friends with whom she talked regularly. She expressed how good she was feeling about her new network.

Another promotora shared how excited one of the caregivers was when sharing that she was finally taking time to relax for a few minutes every day. Such accomplishments were celebrated and recognized.

The final component of the LARREDS model (Figure 1) is dissemination and sustainability. The PODER team has been engaging in various efforts to disseminate our work and has been working with parent‐led organizations that are enthusiastic to offer the *PODER Familiar* program to their constituents. We will continue to seek input from our community partners and promotoras on making the program available to Latinx caregivers and other parents of children with disabilities who might benefit once we analyze the results of our subsequent studies. Two of our community advisory board members represent community agencies interested in adopting *PODER Familiar* and training promotoras to support families of children with IDD. We are also partnering with a local community agency that trains Latinx promotoras to promote the health of the Latinx immigrant community. Yet, they have not worked with families of children with IDD, and we plan to train their promotoras using our model and by developing training the trainers workshops. Barrera et al. (2017) argue that important dilemmas of culturally tailored interventions include improving interventions' reach, engagement, and sustainability. It is anticipated that promotora‐delivered interventions following the LARREDS model described here will address these critical challenges. Throughout the process of applying the LARREDS model through PODER Familiar, caregivers consistently alluded to the benefits of having the intervention delivered by a promotora—a caregiver like them of a child with IDD. Engagement and sustainability of evidence‐based interventions adapted to diverse migrant populations continue to be challenging, and future research is needed on effective strategies for sustaining interventions after external funding ends (Castro & Yasui, 2017).

This section presented the LARREDS Model and how it was co‐created with our promotoras. This description included contributions from the promotoras and demonstrated the power of interventions that engage individuals with lived experience as interventionists. The promotoras, who are also Latinx mothers of children with IDD, helped us to think deeply about the cultural and contextual issues faced by caregivers in the study. They take their role seriously and deepen our understanding of the caregivers' health and wellness experiences.

## DISCUSSION

With growing ethnic diversity in U.S. society, partly due to immigration, cultural tailoring of health promotion interventions has become a public health imperative (Joo & Liu, 2021). The LARREDS model described in this paper can serve as a guide to achieving this imperative. This paper fills a critical gap in the literature by detailing the model and its various dimensions and describing how community voices were engaged throughout model development and refinement. As suggested by Joo and Liu's (2021) scoping review, developing culturally tailored interventions remains opaque, creating a need for practical models and guidelines that can be applied to diverse communities and settings. To this end, the LARREDS model and *PODER Familiar* exemplar offer multiple insights and strategies.

First, the LARREDS model prompts attention to surface and deep structure elements in developing culturally tailored interventions as recommended by Griffith et al. (2024) and Resnicow et al. (2009). ‘Language and linguistic preferences’ (surface elements) and incorporating cultural ‘values and ways of thinking and doing’ and generational differences (deep elements) are critical dimensions of the LARREDS model and must be considered from the inception of an intervention. When developing *PODER Familiar*, we incorporated these elements using a strengths‐focused approach. Consistent with the values of community psychology, social work, and disability studies, intervention development should be driven by a strength‐focused emphasis on cultural values and preferences rather than focusing on disparities and deficits for the target community (Griffith et al., 2024; Suarez‐Balcazar, 2020). Another strength of the LARREDS model is the in‐built flexibility embedded within the dimensions of ‘accessibility’ and ‘delivery and learning styles.’ This flexibility allowed for the *PODER Familiar* intervention to be standardized for all participants and, simultaneously, customizable for the varying access and learning needs of caregivers and children with IDD. These issues are fundamental when designing health promotion interventions targeting individuals with IDD and their families.

The LARREDS model and our experience with *PODER Familiar* also offer essential insights for tailoring interventions for immigrant communities where generational differences would be an important consideration. Thus, we needed to include a variety of metaphors, examples, and recommendations that would hold meaning and relevance for different generations.

An essential contribution of this paper is the centering of promotora voices throughout the design, development, and refinement of the *PODER Familiar* intervention. Promotoras are ideal change agents as they represent the target community; they share many of the same everyday realities and constraints as intervention recipients.

The eight engagement and retention strategies within the LARREDS model, well developed in the family engagement literature, reflect the shared lived realities between promotoras and participants. Scholars have alluded to the need to include engagement and sustainability strategies to address low retention rates as an essential weakness in studies of culturally tailored interventions (Barrera et al., 2017; Castro & Yasui, 2017; Joo & Liu, 2021).

There are limitations of this work that need further research. We did not report on the effectiveness of the components depicted in the LARREDS model, including the engagement strategies. Future research needs to focus on the effectiveness and validity of the model components and engagement strategies. We also need to document the model's effectiveness in developing the intervention. Future research is needed to document both, the effectiveness of the model and the impact of PODER Familiar. While a process and outcomes evaluation were beyond the scope of this paper, the PODER team is currently documenting process measures such as recruitment, retention, fidelity, and acceptability of *PODER Familiar*, as well as its preliminary efficacy during a pilot testing phase followed by a randomized control trial.

One challenge we experienced, was depicting the heterogeneous Latinx population. Future research needs to evaluate the extent to which the *PODER Familiar* intervention applies to diverse Latinx populations. Per the promotoras feedback, another potential challenge is that the promotora becomes part of the support system of the caregiver, and once the intervention ends, the caregiver is short of that support system. Future research should explore strategies to connect Latinx immigrant caregivers to existing support systems and parent‐to‐parent networks.

The work described here has important implications for advancing intervention development with diverse migrant populations. Often, interventions developed for the general populations are imposed on immigrant groups, with little to no attention to deep structure adaptations (Abraham et al., 2024). Unfortunately, this practice perpetuates oppression and exacerbates disparities. To advance immigrant and refugee rights, as well as promote empowerment, health equity interventions need to be culturally relevant and tailored to the community of interest (Abraham et al., 2024). This is even more critical when promoting the wellbeing and health equity of immigrant families of children with IDD, given the disparities they experience and the lack of work in this area in the community psychology field and other related fields (Suarez‐Balcazar et al., 2023). As described in the model, attending to the delivery and learning styles is critical. Robust literature has documented the power of training promotoras to deliver culturally tailored health promotion interventions to immigrant and refugee populations (see Abraham et al., 2024). More research is needed in this area to explore further the impact of promotoras on promoting the health equity of migrant populations.

The conceptual model and case study of *PODER Familiar* provide a critical reference for other researchers and practitioners interested in co‐developing culturally tailored interventions for diverse families of children with IDD. Bringing community voices to the research process is a benefit of more active promotora and community involvement in the research process. Promotoras and our advisory boards contributed important insights and perspectives to the intervention that enhanced *PODER Familiar*'s social and cultural appropriateness. Future research needs to explore the adaptation of the promotoras model to diverse migrant populations. Implementing culturally tailored interventions will contribute to the amelioration of health inequities that disproportionately impact Latinx children with IDD and their families.

As Castro and Yasui (2017) stated, limited studies are available on the best approaches to integrating and adapting evidence‐based interventions within educational, social services, and health care agencies. There is a need for rigorous implementation research when adapting and designing interventions (Chambers & Norton, 2016), and more so when interventions are tested with diverse populations (Castro & Yasui, 2017), including immigrant populations. The LARREDS model illustrated here through the *PODER Familiar* intervention is an essential contribution to this literature.

To conclude, in this paper, we described the co‐creation of an intervention for Latinx families of children with IDD and a model that may be useful for developing culturally tailoring interventions more broadly. The model emerged from the intervention development and piloting process and was significantly strengthened by the contributions of our project promotoras, who have shared lived experiences with the caregivers in our study.

## CONFLICT OF INTEREST STATEMENT

The authors declare no conflicts of interest.
